# Apoptosis in megakaryocytes: Safeguard and threat for thrombopoiesis

**DOI:** 10.3389/fimmu.2022.1025945

**Published:** 2023-01-04

**Authors:** Shuo Yang, Long Wang, Yuesong Wu, Anguo Wu, Feihong Huang, Xiaoqin Tang, Fahsai Kantawong, Songyot Anuchapreeda, Dalian Qin, Qibing Mei, Jianping Chen, Xinwu Huang, Chunxiang Zhang, Jianming Wu

**Affiliations:** ^1^ School of Pharmacy, Southwest Medical University, Luzhou, China; ^2^ Institute of Cardiovascular Research, the Key Laboratory of Medical Electrophysiology, Ministry of Education of China, Medical Key Laboratory for Drug Discovery and Druggability Evaluation of Sichuan Province, Luzhou Key Laboratory of Activity Screening and Druggability Evaluation for Chinese Materia Medica, Luzhou, China; ^3^ Department of Medical Technology, Faculty of Associated Medical Sciences, Chiang Mai University, Chiang Mai, Thailand; ^4^ School of Chinese Medicine, The University of Hong Kong, Hong Kong, Hong Kong SAR, China; ^5^ School of Basic Medical Sciences, Southwest Medical University, Luzhou, China

**Keywords:** megakaryocytopoiesis, thrombopoiesis, apoptosis, apoptosis-related molecules, autophagy

## Abstract

Platelets, generated from precursor megakaryocytes (MKs), are central mediators of hemostasis and thrombosis. The process of thrombopoiesis is extremely complex, regulated by multiple factors, and related to many cellular events including apoptosis. However, the role of apoptosis in thrombopoiesis has been controversial for many years. Some researchers believe that apoptosis is an ally of thrombopoiesis and platelets production is apoptosis-dependent, while others have suggested that apoptosis is dispensable for thrombopoiesis, and is even inhibited during this process. In this review, we will focus on this conflict, discuss the relationship between megakaryocytopoiesis, thrombopoiesis and apoptosis. In addition, we also consider why such a vast number of studies draw opposite conclusions of the role of apoptosis in thrombopoiesis, and try to figure out the truth behind the mystery. This review provides more comprehensive insights into the relationship between megakaryocytopoiesis, thrombopoiesis, and apoptosis and finds some clues for the possible pathological mechanisms of platelet disorders caused by abnormal apoptosis.

## 1 Introduction

Platelets are small anucleate cells derived from mature megakaryocytes (MKs) in the bone marrow and have the appearance of oval discs approximately 2-3 μm in diameter (1). In humans, MKs in the bone marrow produce approximately 100 billion platelets per day (2), and normal physiological platelet counts ranging from 150 to 450 × 10^9^/L of blood (3). Platelets are crucial for thrombosis, hemostasis, wound healing and inflammation (4, 5). Nevertheless, excessive production and activation of platelets can lead to the development of atherosclerosis, myocardial infarction, and ischemia of peripheral limbs, which are all harmful to the body (6). Therefore, the stability of the circulating platelet count is vital for maintaining health, and this is achieved through a dynamic balance between platelet production and consumption/clearance, which must be strictly regulated to maintain the total platelet count within a narrow physiological range (7). Thus, understanding the mechanisms underlying the regulation of thrombopoiesis is important.

Apoptosis plays an important role in the normal growth and development of multicellular organisms, maintenance of self-stable balance, resistance to interference by various external factors, embryonic development, hematopoiesis, immune responses, and aging (8). Multiple apoptotic pathways work synergistically to ensure that multicellular organisms remain healthy and that defective cells are removed from the body. The development, survival, function, and turnover of each cell type, ranging from stem cells to terminally differentiated effector cells, is promoted and controlled by apoptotic pathways (9, 10).

Despite our knowledge of thrombopoiesis, what triggers MKs to produce and release platelets remains unclear. While thrombopoiesis is closely related to apoptosis, this relationship has been questioned in recent years. In this review, we provide an overview of the subtle relationships among megakaryocytopoiesis, thrombopoiesis, and apoptosis, and we summarize the role of apoptotic pathways and their related molecules in regulating thrombopoiesis. In addition, we discuss whether there may be other, more limited, or specific apoptosis-like processes involved in thrombopoiesis. Characterization of the molecules that regulate this process will provide more comprehensive insights into the mechanisms involved in thrombopoiesis and apoptosis and may lead to a better understanding of the mechanisms underlying congenital or acquired thrombocytopenia.

## 2 Overview of thrombopoiesis

**Figure 1:**
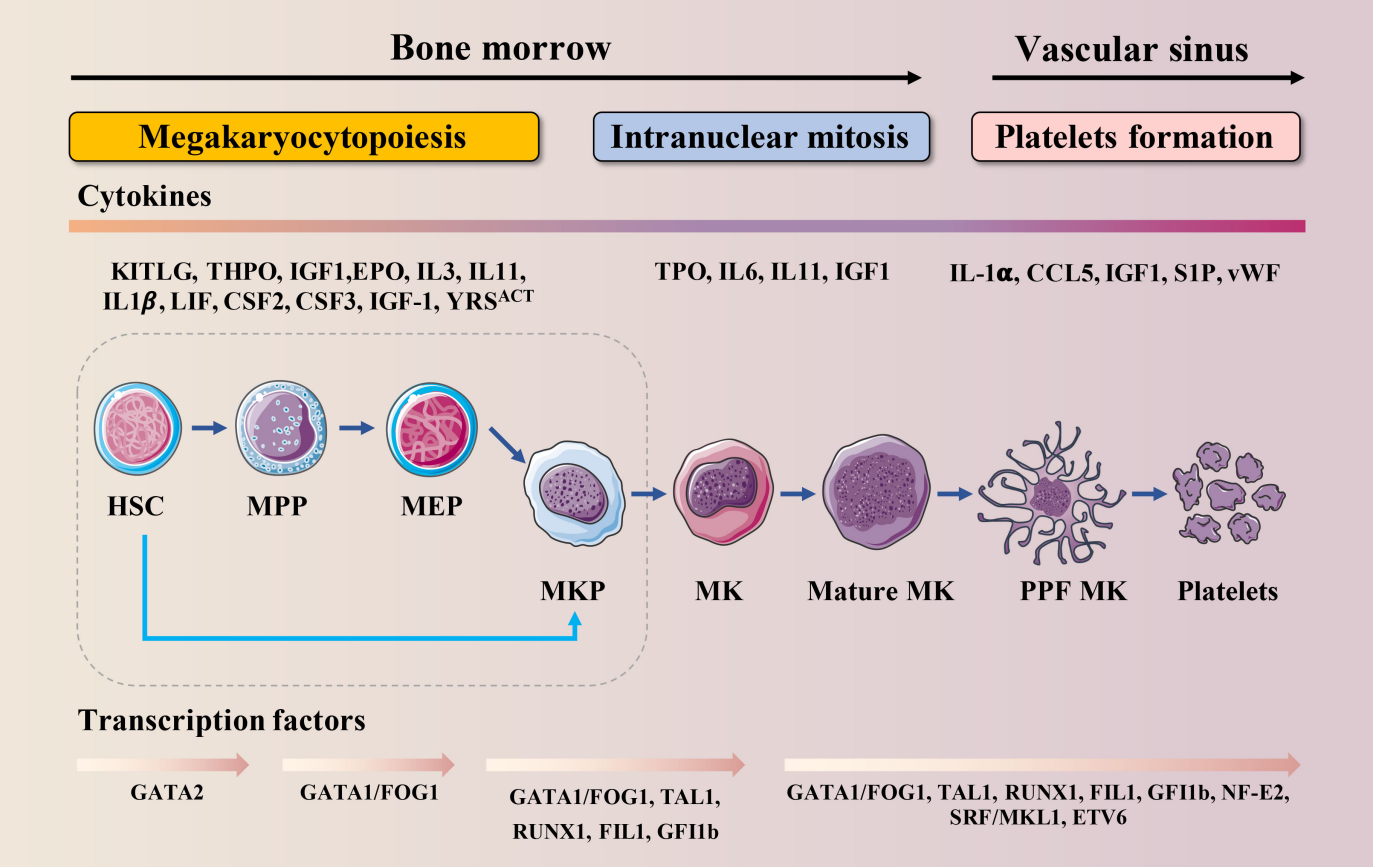
Schematic illustration of MK lineage commitment, intranuclear mitosis and maturation and platelet formation. This drawing depicts the essential developmental stages of thrombopoiesis. MKs originate from HSCs in the bone marrow. Developing MKs undergo intranuclear mitosis to increase chromosome number, then mature before migrating to the vascular sinus where they extend proplatelets or migrate themselves across the endothelial cell barrier into the vascular sinus. These events require the interaction of various cytokines with their receptors and are regulated by multiple transcription factors.

Thrombopoiesis, the process by which MKs mature to form and release functional platelets into the circulating blood (11), can be divided into three sequential stages (**Figure 1**). The first stage is megakaryocytopoiesis, the process by which MKs are derived from bone marrow hematopoietic stem cells (HSCs), the largest cells in the bone marrow, with a diameter of 40–70 μm and up to 100 μm, and comprising <0.05% of the total number of nucleated cells in the bone marrow (12). As per the progressive hematopoietic differentiation model, HSCs asymmetrically differentiate into multipotent progenitor cells (MPPs) that gradually lose their self-renewal ability and pluripotency to generate megakaryocytic/erythroid progenitors (MEPs). MEPs can differentiate into unipotent megakaryocyte progenitors and megakaryocyte precursor cells (MKPs), which then mature into MKs (13, 14). However, this model has been challenged over the past decade. Recently, HSCs with a differentiation bias toward the MK lineage have been identified. These bypass MPPs generation and directly generate unipotent MK-restricted progenitors when mature cells must be rapidly replenished (15–17). MK-restricted progenitor plays a critical role in response to acute platelet demand (18, 19). In the second stage, MKs undergo endomitosis to form polyploids (20). During this stage, MKs are filled with cytoskeletal proteins, platelet-specific granules, and sufficient membrane and undergo an increase in nuclear proliferation and cytoplasmic enlargement. The completion of this process produces many highly differentiated and mature polyploid MKs with large cytoplasmic areas. On the third stage, mature MKs undergo plenty of biomolecular processes, such as caspase activation and extensive cytoskeletal rearrangement, which drive the formation of cytoplasmic extensions called proplatelets (21). These are filled with granules and organelles, and are released as platelets into the blood under the action of blood-flow shear forces (22). This stage is relatively fast and completed within an hour. Demarcated membrane system (DMS),a significant invagination of the plasma membrane, can be observed in the cytoplasm under an electron microscope (23), which divides the cytoplasm of the cells into separate zones defined as platelet territories (24). It takes approximately 5 days for human MKs to complete polyploidization, maturation and platelet release, and approximately 2–3 days for rodent MKs. Once released into the blood, human platelets survive for 7–10 days, whereas rodent platelets survive for 4 to 5 days (25).

Megakaryocytopoiesis and thrombopoiesis are regulated by multiple cytokines, chemokines and transcription factors (TFs) (**Figure 1**). Cytokines and chemokines contribute to megakaryocytopoiesis and thrombopoiesis, ensuring a steady-state of the number of circulating platelets. Megakaryocytopoiesis begins with thrombopoietin (TPO) signaling through its receptor c-MPL, which is expressed at every stage of differentiation from HSCs to platelets (26). TPO binds to c-MPL, leading to the activation of Janus kinase 2 (JAK2). JAK2 phosphorylates c-MPL, leading to the recruitment of a variety of effectors such as signal transducers and activators of transcription (STATs), phosphoinositide-3 kinase (PI3K), and mitogen-activated protein kinase (MAPK) (27). One claim is that the mechanism by which TPO stimulates megakaryocytopoiesis is *via* its ability to prevent apoptosis (28). Once MKs reach a critical maturation stage, these factors may no longer be protected, and apoptosis may occur. Besides TPO, interleukin (IL)-3 (29), stem cell factor (SCF, also known as kit ligand) (30), IL-6 (31), IL-9 (32), IL-11 (31), IL-1-alpha (33), Flt-3 Ligand (FL) (34), Tyrosyl-tRNA synthetase (YRS^ACT^) (35), chemokine (C-C motif) ligand 5 (CCL5, also known as RANTES) (36), insulin-like growth factor-1 (IGF-1) (37), stromal cell-derived factor-1 (SDF-1), and fibroblast growth factor 4 (FGF-4) (38) are also essential for megakaryocytopoiesis and thrombopoiesis. Among these, the actions of IL-1, YRS^ACT^, CCL5, and IGF-1 are independent of TPO.

At the intracellular level, several TFs, cofactors, and chromatin modifiers control the lineage-specific transcriptional programs required for megakaryocytopoiesis and thrombopoiesis by activating or repressing gene expression (**Figure 1**). GATA-binding protein 1 (GATA−1), Friend of GATA−1 (FOG−1), Friend leukemia virus integration 1 (FLI1), Runt-related transcription factor 1 (RUNX1), and Nuclear factor erythroid 2 (NF-E2) are hematopoietic TFs that are crucial for this process (13, 39). Among them, the interaction between GATA-1 and FOG-1 is necessary for MK differentiation and maturation (40). The size of MK in the bone marrow of GATA-1 knockout mice is reduced, and there are morphological changes such as reduced nuclear lobulation, insufficient cytoplasm, and dysplasia of the membrane; the number and function of platelets are also affected (41, 42). FOG-1 is a multi-type zinc finger protein that interacts with GATA-1 to induce the maturation and differentiation of megakaryocytic lineage (43). FLI1 and GATA-1 activate specific genes that regulate late-stage MKPs (44). FLI1 is an E26 transformation-specific pro-oncogene domain transcription factor. *In vitro* studies indicate that FLI1 overexpression promotes the MK differentiation of K562 erythroleukemic cells (45). Homozygous FLI1 mutants show defective megakaryocytopoiesis and cerebrospinal fluid hemorrhage, followed by death at embryonic day 11.5 (46). In humans, hemizygous deletion of FLI1 leads to Paris-Trousseau/Jacobsen syndrome, an acute bleeding disorder (47). RUNX1 is critical for definitive hematopoietic myeloid differentiation (48). RUNX1-deficient MKs are small and poorly lobulated and show a marked reduction in ploidy (49). Previous studies have shown that inducible deletion of RUNX1 in adult mice leads to a rapid and sustained five-fold decrease in peripheral platelet counts (50, 51). NF-E2, a heterodimer composed of p45 and p18, is the master regulator of thrombopoiesis (52). It has been reported that MKs in NF-E2 knockout mice are mature and intact but fail to produce platelets (53). Therefore, NF-E2 knockout mice develop fatal thrombocytopenia due to a lack of circulating platelets and die from bleeding (54).

## 3 Apoptosis and its signaling pathways

### 3.1 Overview of apoptosis

**Figure 2:**
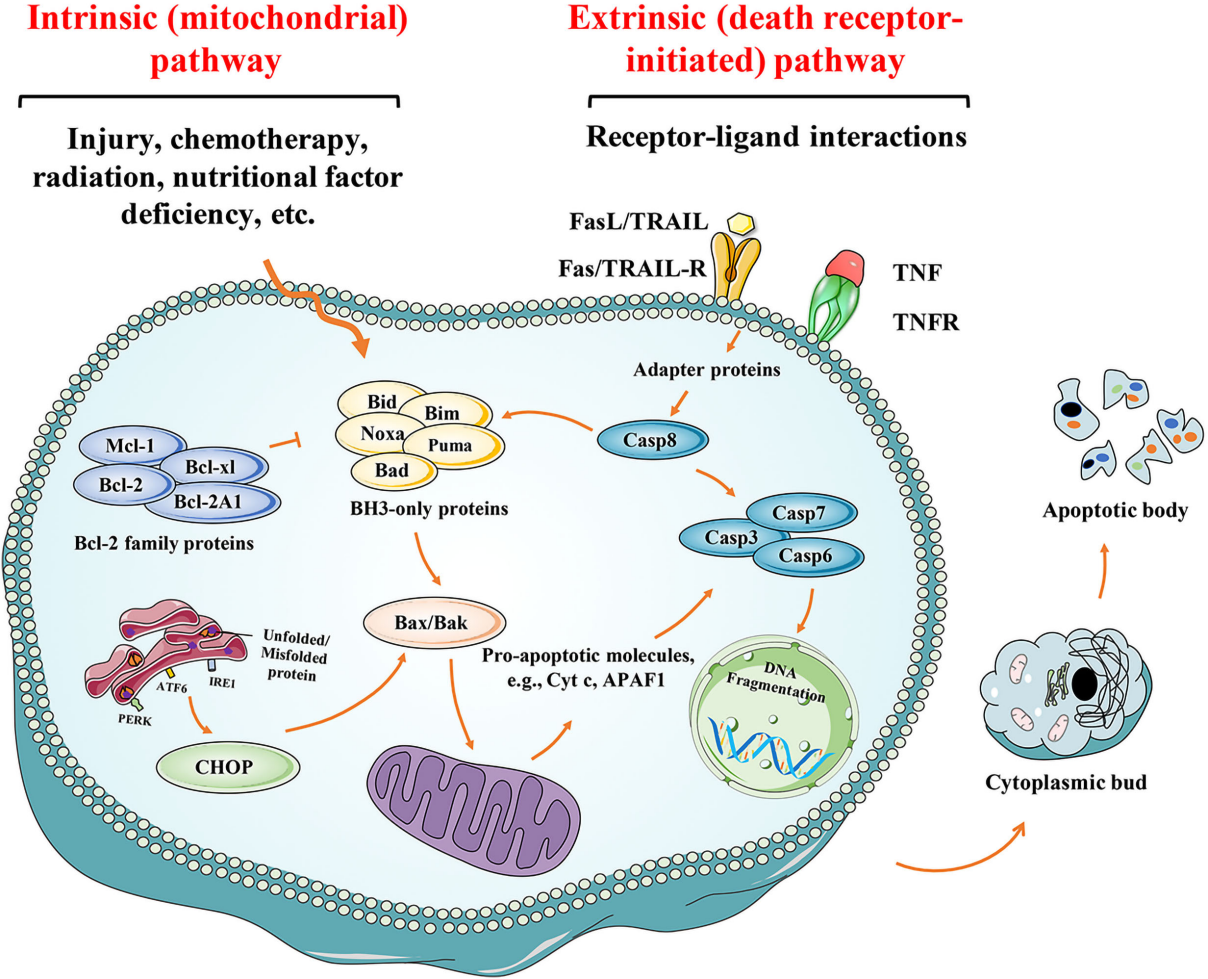
Molecular mechanisms of apoptosis pathway activation. The intrinsic pathway of apoptosis is initiated by the cell itself in response to damage, such as injury, chemotherapy, radiation and nutritional factor deficiency. Cell stresses engage Bcl-2 homology domain 3 (BH3)-only protein activation, leading to the activation of Bak and Bax that triggers MOMP. Anti-apoptotic Bcl-2 family proteins counteract BH3-induced activation of Bak and Bax. Following MOMP, mitochondrial intermembrane space proteins such as second mitochondria-derived activator of caspases (SMAC) and cytochrome C are released into the cytosol. Cytochrome C interacts with apoptotic protease activating factor 1 (APAF1), triggering apoptosome assembly. In the extrinsic apoptotic pathway, upon binding to their cognate ligand, death receptors such as DR2, DR4 and DR5 can activate initiator caspases (caspase-8 and caspase-10) through dimerization mediated by adaptor proteins such as FADD. ERS-induced apoptosis can be regarded as the part of the mitochondrial apoptosis pathway. When ERS occurs, cells initiate an adaptive response called the UPR mainly mediated by the kinase PERK, triggering the mitochondrial apoptotic pathway. Both pathways converge when caspase 3 (executioner caspase) is activated, resulting in formation of apoptotic bodies, eventually leading to cell death.

The word “apoptosis” originates from a Greek word that means “falling off,” i.e., leaves falling from a tree, a natural phenomenon (55), and was originally coined by Kerr et al. in 1972 to describe a morphologically distinct mechanism of “cell deletion” (56). Apoptosis is also known as programmed cell death (or colloquially, “cellular suicide”). During apoptosis, cells cease growth and division, ultimately resulting in controlled cell death. The cell contents do not spill into the surrounding environment. Instead, the unwanted cells and their contents are removed quickly without causing inflammation. Apoptosis has been clearly defined at the biochemical level and is characterized by enzyme-dependent biochemical processes and several specific morphological changes to the cell structure (55). Morphologically, the apoptotic cells are characterized by membrane crinkling and depression, chromatin condensation and marginalization, nucleus shrinkage, nucleus fragmentation, cell membrane blistering and wrapping around the cytoplasm and nucleus in broken pieces, forming apoptotic vesicles (57). The initiation of apoptosis is dependent on the activation of a series of cysteine aspartic proteases known as caspases. The caspase cascade reaction appears to be irreversible and the process eventually leads to cell death once initiated. There are two categories of caspases: initiator and effector caspases (58). Once the body detects cell damage, inactive procaspases (caspases 8 and 9) are activated, which in turn activate the effector caspases (caspases 3, 6 and 7) (59). The activation of effector caspases initiates a cascade of events that results in DNA fragmentation due to the activation of endonucleases, destruction of the nuclear proteins and cytoskeleton, crosslinking of proteins, the expression of ligands for phagocytic cells, and the formation of apoptotic bodies (60) (**Figure 2**).

The process of apoptosis is highly conserved within multicellular organisms and is genetically controlled (61). Eukaryotic cells undergo apoptosis mainly through the mitochondria-mediated intrinsic apoptosis pathway, the death receptor-mediated extrinsic apoptosis pathway, and the endoplasmic reticulum stress (ERS)-mediated apoptosis pathway (**Figure 2**). The cell can initiate apoptosis in itself when it detects damage *via* several intracellular sensors, and this process is known as the intrinsic pathway (62). Alternatively, the extrinsic apoptosis pathway is initiated by the interaction between immune and damaged cells (63). Although these two pathways are different, they share the same processes (activated by the caspase family) to implement apoptosis. In addition to these two classical apoptotic pathways, ERS can also cause apoptosis. ERS is mainly regulated by Bcl-2 family-related pro-apoptotic protein (such as Bax and Bak) and/or anti-apoptotic protein (such as Bcl-2), regulation of Ca^2+^ in the endoplasmic reticulum (ER), and other pathways that lead to cell apoptosis (64). Therefore, ERS-induced apoptosis can be regarded as the part of the mitochondrial apoptosis pathway. Multiple apoptotic pathways work synergistically to ensure that multicellular organisms remain healthy and that defective cells are removed from the body. However, the failure to regulate apoptosis can result in numerous disease conditions (**Figure 2**).

### 3.2 Intrinsic (or mitochondrial) apoptosis pathway

The intrinsic apoptosis pathway is centered on the mitochondria, dependent on the factors released from the mitochondria, and is initiated either by positive or negative stimuli (65). When cells are exposed to various stimulating factors, such as hypoxia, oncogene activation, DNA damage, and lack of cytotropic factors, homeostasis of the mitochondrial membrane is destroyed, and the permeability of the mitochondrial membrane increases. The pro-apoptotic factors in the mitochondrial membrane are released into the cytoplasm, activating the intrinsic apoptotic pathway, and ultimately causing cell death (66).

The intrinsic pathway is controlled by the Bcl-2 family of proteins, which comprise three subsets: the pro-survival proteins (Bcl-2, Bcl-xl, Bcl-w, Mcl-1and A1), the multi-domain killers (Bak and Bax), and the “BH3-only” death initiators (Bid and Bim) (67). Additionally, pro-apoptotic factors include cytochrome C (Cyt c), apoptosis-inducing factor (AIF), cysteine aspartate proteinase activator (Smac/Diablo), and apoptotic protease activating factor-1 (Apaf-1), etc (68). To effectively maintain blood homeostasis, the apoptotic process is tightly controlled by the Bcl-2 family members, which play a role in promoting or inhibiting apoptosis (63). In healthy cells, pro-survival proteins (e.g. Bcl-2, Bcl-cl, Mcl-1, Bcl-w and BFL-1/A1) inhibit the activation of Bak and Bax (69). Without pro-survival signals, inactive pro-apoptotic molecules, such as Bak and Bax, become active and initiate apoptosis. Pro-apoptotic signals induce BH3-only proteins, which overwhelm pro-survival activity and directly activate Bak/Bax. Activation of Bak/Bax induces mitochondrial outer membrane permeabilization (MOMP), causing the pro-apoptotic proteins, including Cyt c, Smac/Diablo, and HtrA2/OMI, to leak out from the mitochondria into the cytoplasm. Cyt c can change the conformation of Apaf-1 by binding to it and exposing the nucleotide-binding and oligomeric domains that can, in turn, bind deoxyadenosine triphosphate (dATP) (70). This subsequently leads to additional conformational changes in Apaf-1, exposing its caspase recruitment domain (CARD) and oligomerization domains, thus enabling several Apaf-1 molecules to assemble into a complex called an apoptosome. Apoptosomes recruit procaspase 9 *via* the exposed CARD domains and activate them to form caspase 9. Caspase 9 is the initial caspase that controls the intrinsic apoptosis pathway *via* the proteolytic activation of the effector caspases 3 and 7. These effector caspases cleave various protein substrates to execute cell destruction (71). The latter step is the “point of no return”, which triggers the outflow of apoptotic factors and ultimately leads to apoptosis (**Figure 2**).

### 3.3 Extrinsic apoptosis pathway

In contrast to the intrinsic apoptosis pathway, the extrinsic pathway, also known as the death receptor pathway of apoptosis, is activated by a more limited number of ligands that interact with transmembrane receptors (72). For instance, death ligands produced by natural killer (NK) cells or the patrolling macrophages bind to death receptors (DRs) in the target cell membrane for activating procaspase 8 and generating caspase 8 (73).

DRs are characterized by the presence of a cytoplasmic domain called the death domain, comprising about 80 amino acids. The DR family consists of more than 20 proteins with a wide range of biological functions and are members of the tumor necrosis factor (TNF) receptor gene superfamily (74). DRs play a key role in transmitting death signals from the cell surface to intracellular signaling pathways and regulate cell death and survival, differentiation, and immune regulation (75). Members of the TNF receptor family share similar cysteine-rich extracellular domains (76). The best-characterized DRs include Death receptor 1 (DR1) (also known as TNF-R1, CD120a, p55), DR2 (Fas, CD95 or Apo-1), DR3 (Apo-3, TRAMP, LARD or TNFRSF25 (TNF receptor superfamily 25)), DR4 (TNF-related apoptosis-inducing ligand (TRAIL)-R1 or Apo-2), DR5 (TRAIL-R2 or TRICK2) and DR6 (TNF receptor superfamily member 21 (TNFRSF21)) (77).

The binding of TNF-α to DR1 causes trimerization of DR1 to form a death receptor complex. This complex can promote cell survival or cell death, depending on other related factors. On the one hand, TRADD leads the activation of the transcription factors such as NF-κB by recruiting RIP and TNF related factor-2 (TRAF2). On the other hand, the binding of TRADD and FADD and procaspase-8 leads to the activation of caspases 3, 6 and 7, resulting in the occurrence of apoptosis either directly or indirectly (78).

DR2 is a type I transmembrane protein that belongs to the TNF receptor family, while FasL is a type II transmembrane protein belonging to the TNF superfamily (79). The DR2 receptor binds to its cognate ligand FasL and recruits DR2-associated death domain protein (FADD) and caspases 8/10 through its intracellular death domain to form a death-inducing signaling complex, which in turn activates other procaspases, ultimately leading to apoptosis (80, 81).

DR4 and DR5 mediated apoptosis are very similar to DR2-mediated apoptosis (82). However, TRAIL induces apoptosis in many human tumor cell lines but not in most normal cells (83). Immune surveillance cells, such as T lymphocytes, B lymphocytes, NK cells, dendritic cells, monocytes and granulocytes, upregulate surface TRAIL expression and/or release soluble TRAIL stored in secretory vesicles upon receiving activation signals (84). Although TRAIL plays a major role in mediating different immune responses, most studies have demonstrated that TRAIL also exhibits a regulatory function in hematopoiesis under both normal and pathophysiological conditions (85). For instance, TRAIL can induce apoptosis of leukemic malignant cells without killing normal cells (86). TRAIL can also promote the differentiation of surviving leukemic cells and normal myeloid precursors into mature monocytoid cells, thereby regulating hematopoiesis (87) (**Figure 2**).

### 3.4 Endoplasmic reticulum stress (ERS)-mediated apoptosis pathway

The endoplasmic reticulum (ER) plays a crucial role in protein metabolism and signal transduction. In fact, there are important mechanisms in ER that ensure high-quality protein folding, which is necessary for cell survival, function, and homeostasis. Exposure to adverse environments perturbs ER homeostasis, leading to misfolding and accumulation of unfolded proteins in the ER lumen, a phenomenon known as ER stress (ERS) (88). The unfolded protein response (UPR), also known as the ERS response (ERSR), is triggered by ERS to rebuild homeostasis or stimulate cell death (89). The UPR constitutes an adaptive anti-apoptotic survival response during exposure to ERS. However, when the UPR fails to restore ER homeostasis under severe or prolonged ERS conditions, ERS mediated apoptosis will occur (90). ERSR is primarily mediated by three sensor proteins residing in the ER, namely the protein kinase RNA-like endoplasmic reticulum kinase (PERK), activating transcription factor 6 alpha (ATF6) and inositol requiring enzyme 1 alpha (IRE1) (91). Integrated signaling downstream of these three sensors can promote pro-apoptotic signaling by stimulating C/EBP homologous protein (CHOP), which downregulates Bcl-2 protein and upregulates Bax protein (92). Bcl-2 and Bax regulate a variety of cellular reactions to ERS by transporting Ca^2+^ into and out of the ER lumen (93). The Ca^2+^ delivered from the ER *via* inositol 1,4,5-trisphosphate receptors (IP3Rs) and ryanodine receptors (RyRs) at ER and mitochondrial contact sites can cause apoptosis, mostly through cytochrome c transfer from the mitochondria (94). In addition, caspase-12 can also induce apoptotic pathways triggered by ERS. Caspase-12 is reported to be the first ER-associated member of the caspase family (95). Depending on the stressors, caspase 12 activation may require either the transfer of caspase 7 from the cytoplasm to the ER membrane or the recruitment of calcium-mediated calpain to the ER surface (96–98). After that, it further triggers the activation of cytoplasmic caspase 3, ultimately leading to apoptosis (99) (**Figure 2**).

### 3.5 ROS and apoptosis

**Figure 3:**
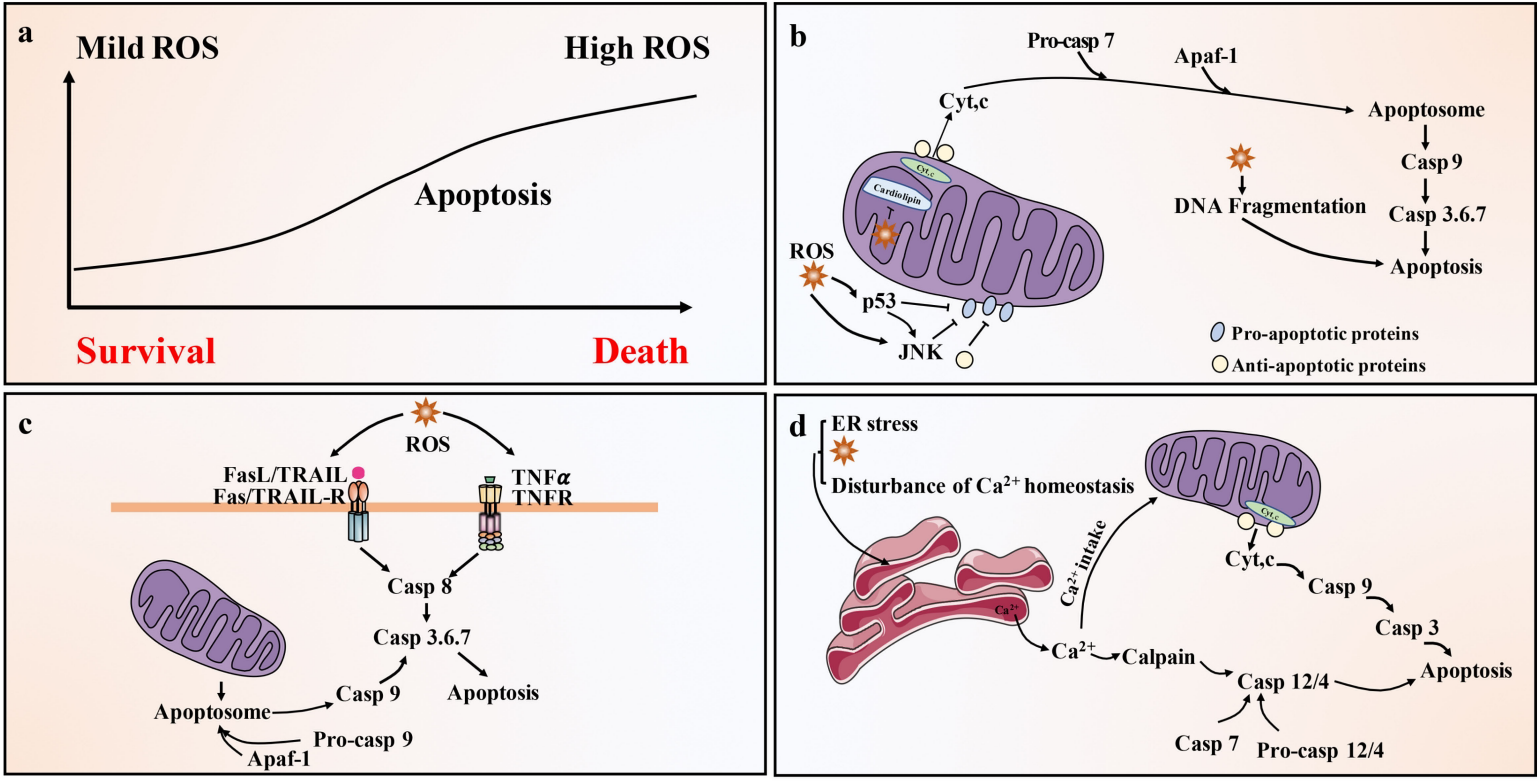
Pathways of ROS-driven apoptotic responses. **(A)** Low level of ROS (such as H2O2) is related to cell survival, but high level of ROS can activate cell death processes, such as apoptosis. **(B)** Activation of the mitochondrial (intrinsic) pathway of apoptosis by ROS. Exogenously or endogenously generated ROS can activate p53 and/or c-JNK, which activate pro-apoptotic Bcl-2 proteins and inhibit anti-apoptotic proteins. In addition, ROS cause mitochondrial damage membrane depolarization and/or opening of Bax/Bak channels on the outer mitochondrial membrane (OMM), allowing AIF, Endo G, Cyt c and Smac/Diablo to be released into the cytoplasm. Cyt c then forms apoptosome complex in the cytoplasm along with Apaf-1 and procaspase-9, leading to caspase 9 activation. Caspase 9 then activates effector caspase, such as caspase 3, ultimately causing apoptosis. **(C)** Activation of the death receptor (extrinsic) pathway of apoptosis by ROS. Death receptor-mediated apoptosis involves the recruitment of the bridging proteins FADD and procaspase-8 or -10 to the cytoplasmic surface of the receptor to form death-inducing signaling complex (DISC). This leads to the activation of caspase-8/-10, which can directly activate caspase-3/-6/-7 and trigger apoptosis. Caspase-8/-10 also cleaves Bid to produce tBid, which blocks the anti-apoptotic activity of Bcl-2 and Bcl-xl, and activates Bax and Bak. This leads to the release of Cyt c and Smac/Diablo and the activation of the mitochondrial pathway of apoptosis. **(D)** ROS activates the ER stress response and cause apoptosis through the ER. Ca^2+^ released from the ER is taken up by the mitochondria, leading to depolarization of the inner mitochondrial membrane (IMM). ER caspase-4/-12 activation by calpain and possibly caspase-7 leads to activation of caspase-9 and caspase-3. Caspase-9 and 3 can be activated through mitochondria-dependent and independent pathways.

Lower levels of ROS (such as H_2_O_2_) are related to cell survival, but higher doses of ROS will activate cell death processes, such as apoptosis (100) (**Figure 3A**). ROS is closely related to apoptosis activation of the mitochondrial pathway. In fact, mitochondria are where most ROS are produced in cells, as a result of leakage in the respiratory electron transport chain (101). Mitochondrial-generated ROS can target nearby structures, leading to disruption of respiratory chain function, further increasing ROS levels, resulting in loss of mitochondrial sites and impaired ATP synthesis, ultimately leading to apoptosis (102). In addition, ROS such as H_2_O_2_ or superoxide can also cause Cyt c release from mitochondria and induce mitochondrial pathway apoptosis (78) (**Figure 3B**). There is also a link between ROS and the death receptor-mediated extrinsic apoptotic pathway. It is widely believed that TNF-derived ROS can inhibit the activation of NF-κB and reduce the survival signal mediated by NF-κB, thereby promoting apoptosis (103). ROS can also lead to activation of JNK, followed by release of Cyt c, activation of caspase 3 and apoptosis (104). ROS can also activate Fas-mediated apoptosis. H_2_O_2_ can upregulate FasL and activate caspase 8 and caspase 8 processed caspase 2, leading to the cleavage of Bid to t-Bid, thereby enhancing mitochondria-induced apoptosis (105). Members of the Nox family are important sources of ROS production (106). Binding of FasL and TNF-α to the corresponding death ligand receptors leads to lipid raft formation, recruitment and activation of Nox and ROS generation. These processes constitute lipid raft derived redox platforms that promote the activation of death receptors for apoptosis (107). ROS is also linked to TRAIL-mediated apoptosis, although the mechanism remains unclear. ROS can up-regulate the expression of TRAIL (**Figure 3C**). It is estimated that approximately 25% of intracellular ROS is generated due to the formation of disulfide bonds during the oxidative folding of proteins in the lumen of the ER (108). The production of ROS, a byproduct of protein oxidation in the ER, also leads to ER stress-mediated apoptosis (109) (**Figure 3D**).

## 4 A relationship between thrombopoiesis and apoptosis

It is long known that thrombopoiesis is closely associated with MKs apoptosis, but a generally accepted unifying hypothesis for the role of apoptosis in thrombopoiesis has remained elusive.

### 4.1 Positive effects of apoptosis on thrombopoiesis

In recent years, the role of the apoptotic pathway in megakaryocytopoiesis and thrombopoiesis has attracted much attention. Considerable evidence has shown that apoptosis is necessary for MKs to produce platelets.

In the late 1990s, researchers believed that a large decrease in platelet count was due to impaired apoptosis, which prevented efficient platelet shedding from MKs. Some researchers have found that apoptosis occurs mainly in mature MKs rather than in immature ones (110), and apoptosis and platelet formation seem to occur simultaneously after MK maturation (111). The kinetics of platelet release is delayed relative to the peak of MK maturation, but it is related to the occurrence of apoptosis (112). In addition, many characteristics of platelet shedding from mature MKs are similar to that of programmed cell death, such as ruffling, blebbing, condensation of the plasma and nuclear membranes, reorganization and disruption of the cytoskeletal architecture, DNA fragmentation, cell shrinkage and packaging of cellular components into vesicles, and formation of apoptotic bodies (113). The first evidence that platelet formation is linked to MK apoptosis was provided by the observation that platelet formation was associated with the cleavage of caspase 3 and caspase 9 in MKs derived from cord blood, bone marrow, and peripheral blood CD34^+^ cells (114). When MKs undergo shedding to produce platelets, procaspase 3 is cleaved into enzymatically-active caspase 3 fragments (115). Local activation of caspase 3 correlates with mitochondrial membrane permeability, leading to the activation of intrinsic pathways. The second line of evidence is provided by the significant reduction of proplatelet and functional platelets by a broad-spectrum caspase inhibitor, zVAD.fmk, indicating that caspase-directed apoptosis plays a role in the formation of proplatelets from MKs and the subsequent production of functional platelets (116). Moreover, MKs also exhibit prominent heterochromatin condensation, a typical early apoptosis feature, at the earliest cytoplasmic projection stage (117). NO has been shown to positively regulate thrombopoiesis by inducing apoptosis of mature MKs through potential pro-apoptotic factors such as TNF-α and IFN-γ (118). Other pro-apoptotic factors, such as TGF-β and SMAD proteins, have likewise been implicated in platelet production (119). Phorbol myristate acetate (PMA), known to induce differentiation of K562 and DAMI cells, provides an ideal model for studying MK differentiation (120–123). A recent study reports that PMA can activate pro-apoptotic proteins such as Bax and Bim while inhibiting the anti-apoptotic Bcl-2 in DAMI cells. In addition, annexin V-positive cells significantly increased in PMA-induced DAMI cells compared to those in the control group, and the cleaved product of poly ADP-ribose polymerase (PARP) was observed (124). Caspase activation and PARP cleavage are known markers of apoptosis (125). Recently, the actin cytoskeleton has been implicated in proplatelet formation (PPF) (126). MKs derived from human umbilical cord blood CD34^+^ cells are treated with the actin polymerization inhibitor latrunculin, resulting in increased ploidy and PPF in cultured CD34^+^-derived MKs. At the same time, increased expression of pro-apoptotic and pro-survival genes is observed, and the effect of actin inhibition on PPF can be blocked by caspase inhibition such as zVAD.fmk or Q-VD-OPh (127). All these findings support an unignorable role of apoptosis in proplatelet formation.

**Figure 4:**
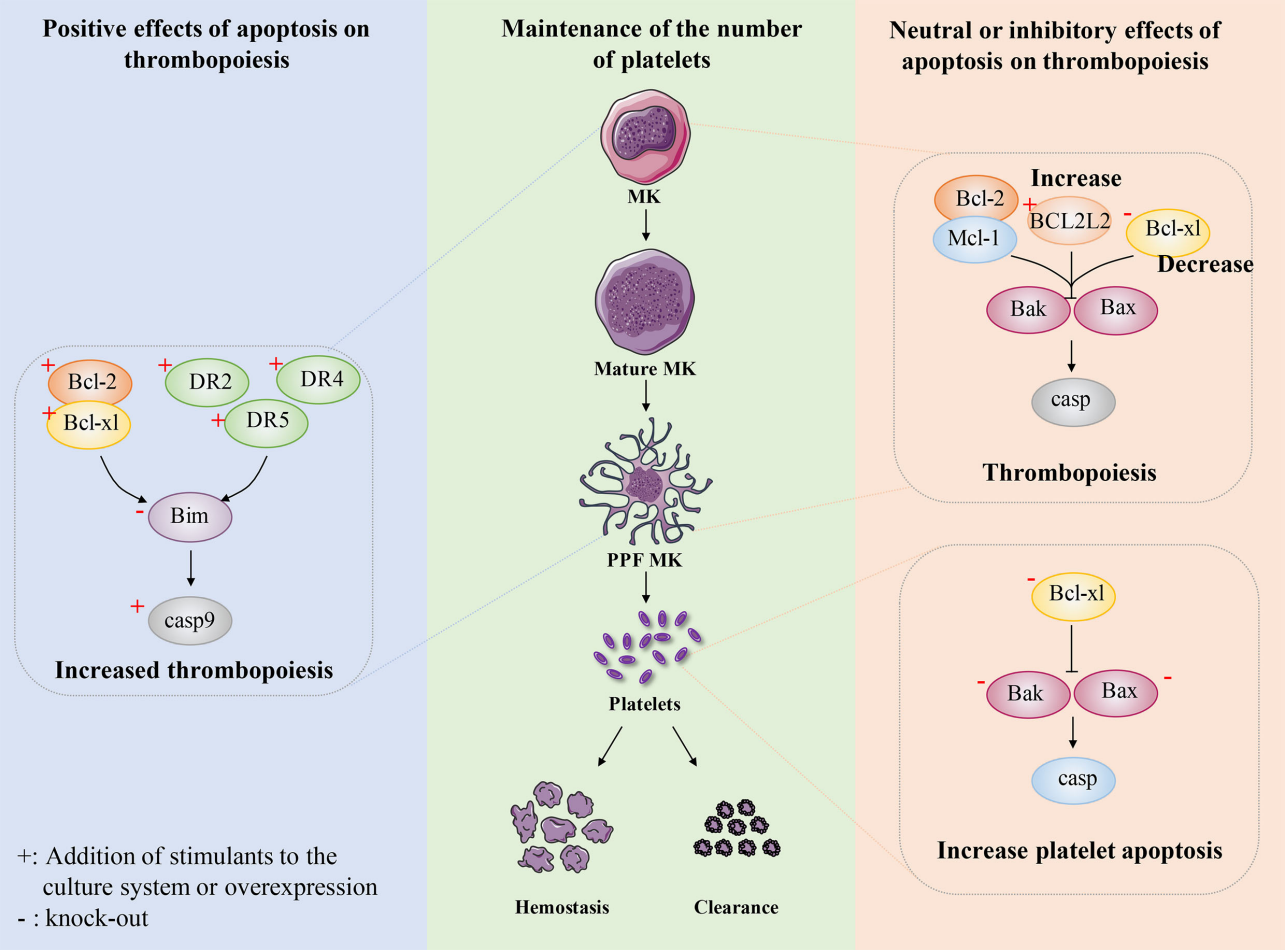
The role of apoptosis in megakaryocyte and platelet biology. Maintaining the number of platelets in a dynamic balance range is very important for normal hemostasis, coagulation and immune function. The number of circulating platelets is closely related to the lineage development of MKs, platelet production and platelet apoptosis. Some researchers believe that platelet production depends on apoptosis. After expressing human anti-apoptotic protein Bcl-2 in mice, or overexpressing anti-apoptotic protein Bcl-xl in mice, or knocking out pro-apoptotic protein Bim in mice, intrinsic apoptosis was inhibited to some extent and the number of platelets decreased. In addition, exogenous stimulation DR2, DR4, DR5, the apoptosis pathway is activated to some extent, which shows the increase of circulating platelets. These results indicate that platelet production depends on apoptosis. Recently, researchers have conducted a relatively systematic study on the development of platelet number-related MK lineage, platelet production and platelet apoptosis. They believe that platelet production does not depend on apoptosis and endogenous apoptosis should be inhibited. The absence of Bcl-2 and Mcl-1 had no effect on platelet count. Overexpression of BCL2L2 inhibits apoptosis to a certain extent, resulting in the failure of PPF. The absence of Bcl-xl results in severe thrombocytopenia, which not only promotes platelet apoptosis and shortens platelet life, but also seriously damages platelet production. Bax/Bak have little effect on platelet production, but after deletion, it results in prolonged platelet life and relative increase in peripheral platelet number. These findings indicate that platelet production do not depend on apoptosis and intrinsic apoptosis should be inhibited.

Bcl-2 family proteins are essential in regulating endogenous apoptosis pathways (67). In the early stage, Ogilvy et al. targeted the expression of the human Bcl-2 gene in mice, and to their surprise, the number of platelets in the blood of the mice was almost half that of normal mice (128). This notion is supported by similar evidence. Bouillet et al. develops a proapoptotic Bim knockout mouse model, which shows only about 50% of normal peripheral platelet counts (129). In addition, the pro-survival protein Bcl-xl overexpression is shown to impede PPF in mice (130). These findings suggest that the normal occurrence of the intrinsic apoptotic pathway is extremely important for maintaining the platelet count (**Figure 4**).

The extrinsic apoptotic pathway is triggered by members of the TNF receptor family, such as DR1 or DR2, which can activate caspase 8 (67). Clarke et al. find that the production of platelet-like particles was markedly increased by treatment with FasL or agonistic Fas antibodies in either Meg-01 cells or primary mouse MKs (116). Furthermore, the amount of PPF is also doubled by the addition of caspase 8 in Meg-01 cells or in the primary mouse MKs culture system (131). Similar results are observed using a novel human bone core explant culture system. The trabecular bone of the human femoral head containing usable bone marrow is surgically removed and observed to constitutionally produce platelets that were positive for genealogy-specific fibrinogen receptors four days later. Moreover, platelet-like particles increase when incubated with the activator Fas antibody but decreased when co-incubated with the broad-spectrum caspase inhibitor zVAD.fmk (131). In addition, after adding recombinant soluble TRAIL to primary CD34^+^ cells, the apoptosis rate of CD34^+^/CD41^dim^ cells significantly increased, which promoted the transformation of MKs into fully mature platelet-producing MKs (132). A decrease in TRAIL expression leads to decreased apoptosis in MKs, resulting in impaired platelet production (133). The above findings demonstrate that extrinsic apoptosis regulate the development and maturation of MKs and the production of platelets (**Figure 4**). Although it has reported that FasL- and TRAIL-deficient mice develop fatal autoimmune thrombocytopenia (134), it can be explained that FasL- and TRAIL-deficient mice suffer from severe lymphoproliferative disease, dysregulation of lymphocyte homeostasis leads to antiplatelet IgM and IgG production, which results in thrombocytopenia (133). This does not seem to conflict with previous research.

**Figure 5:**
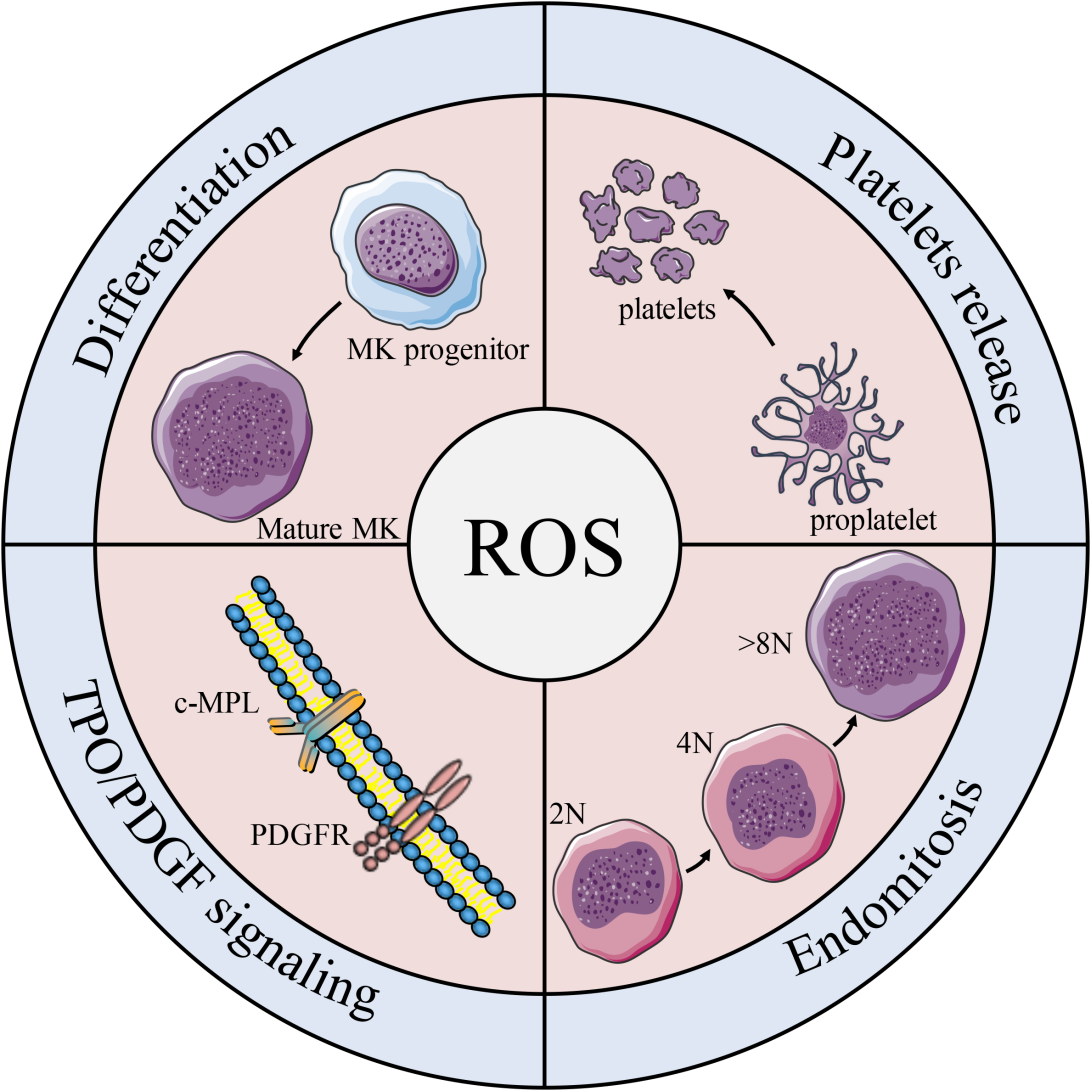
Effects of ROS on platelet formation. Schematic diagram of the effects of ROS on MK biology and platelet production. ROS participates in the differentiation of MK progenitors into MKs, and plays an important role in regulating c-MPL/PDGFR pathway, MK mitosis and platelet release.

Studies have shown that both oxygen tension and ROS levels are involved in MK development, platelet production, and platelet activation. Mature MKs release platelets into the sinuses in the regions near the bone marrow or pulmonary capillaries, areas that contain high levels of O_2_ (135). In the process of endomitosis during the development of MKs, TPO-induced cells are larger and have higher ploidy at higher O_2_ tension (136). Earlier studies on platelet production show that peripheral blood CD34^+^ produce more CD41^+^ MKs under higher oxygen partial pressure, which is associated with increased expression of the MK mature-specific transcription factor GATA-1 and NF-E2 (137). It is worth noting that ROS are located upstream of the signaling cascades required for MK proliferation and differentiation (136). Moreover, the effect of ROS on the mitogen-activated protein kinase/extracellular signal-regulated kinase (MEK)-extracellular signal-regulated kinase (ERK) 1/2 pathway may be a possible mechanism underlying MK differentiation. As previously described, NO promotes MK maturation and platelet production *via* activation of apoptosis. Researchers have also find that O_2_ reacts with NO to form ONOO^-^, a ROS (138) that can activate upstream effectors of ERK, such as MAPK/ERK (139), which significantly enhances thrombopoiesis in the final stage of MK development (140). Furthermore, 48 h after PMA intervention in DAMI cells, ROS production increased by approximately 3-fold, and the cell mitochondrial membrane potential (depolarized mitochondrial membrane) also increased (124). In addition, a decrease in MK apoptosis and platelet production was observed along with a decrease in total ROS levels in the cells (141) (**Figure 5**).Taken together, these results suggest that the apoptosis has a positive effect on thrombopoiesis (**Figure 4**).

### 4.2 Neutral or inhibitory effects of apoptosis on thrombopoiesis

Although over-expression of some anti-apoptotic protein (such as Bcl-2) or knocking out some pro-apoptotic protein (such as Bim) in the apoptotic pathway can reduce the number of platelets, and stimulation of exogenous apoptotic pathway can also promote thrombopoiesis, these results are not completely convincing. Recent studies have made a more in-depth report on the role of apoptosis in the steady maintenance of platelet number. Homeostatic maintenance of platelet count is not only about thrombopoiesis, but platelet clearance/lifespan should also be considered (142). A wide range of apoptotic pathways not only have a strong regulatory role in megakaryopoiesis and platelet production, but also play a very important role in platelet apoptosis (143). The apoptosis of platelets is considered to be the main physiological mechanism to regulate the lifespan of platelets (144). The reported factors affecting platelet apoptosis include apoptosis induced by chemical stimulation and high shear stress, as well as apoptosis caused by platelet aging (145).

Surprisingly, recent studies have reported that platelet production is independent of the intrinsic and extrinsic apoptotic pathways of MKs and that the intrinsic apoptotic pathway should be inhibited during platelet formation. A series of mice with gene deletion related to intrinsic apoptosis pathway were constructed, and were used to explore the relationship between intrinsic apoptosis pathway and platelet production or apoptosis. Mice with MK lineage deficient in Bcl-2 exhibited normal platelet production and platelet lifespan. Deletion of Bcl-2 and Mcl-1 in megakaryocytes had no significantly effects on platelet production or platelet survival. These data suggest that Bcl-2 and Mcl-1 may be dispensable for megakaryocyte and platelet development and survival (146) (**Figure 4**).

Recently, apoptotic regulators of human cord blood-derived (CB)-MK cultures have been reported. BCL2L2 is a member of the anti-apoptosis factor Bcl 2 family, which mainly encodes Bcl-W (147). Krawiec et al. found that overexpression of BCL2L2 resulted in a significant decrease in the percentage of CB-derived MK annexin V^+^ CD41a^+^ MK, while induced a significant 58% increase in PPF MK (148). Moreover, they also found BCL2L2 mRNA levels in platelet were positively correlated with platelet counts by analyzing the genome-wide platelet gene expression profiling of 154 healthy individuals. This finding is consistent with the observed increase in platelet-like granules in MKs derived from cultured overexpressed BCL2L2. Thus, these data suggest that BCL2L2 inhibits apoptosis of cultured MKs, promotes PPK, and correlates with platelet count (**Figure 4**).

Bcl-xl is a survival-promoting factor in the intrinsic apoptotic pathway which can inhibit Bak/Bax. The researchers constructed mice with MK-specific deletion of Bcl-xl to explore the effect of Bcl-xl on platelet count. The number of platelets in knockout mice is only about 2% of normal mice, while other hematopoietic lineages are not affected (149). Moreover, the mice exhibited highly abnormal platelets, with a doubling of mean platelet volume (MPV) and a significantly higher platelet distribution width (PDW). Although it has also been shown that Bcl-xl deficiency induces platelet apoptosis and thus affects platelet lifespan, mathematical modeling of productivity further supports the concept that shortened platelet lifespan cannot explain the severity of thrombocytopenia (150). Thus, the study further found that the mice with MK-specific deletion of Bcl-xl had a slightly increased number of MK progenitors and MKs. However, the mice still exhibited severe thrombocytopenia, which makes us believe that platelet production is closely related to Bcl-xl. They also found that the extensions of MKs in Bcl-xl deleted mice were fewer, shorter and less detailed, and these MKs died soon after forming short extensions. In addition, MKs in Bcl-xl deleted mice showed increased phosphatidylserine (PS) exposure, and caspase-3/7 activity, and decreased ATP levels (149). This suggests that the failure of PPF in Bcl-xl deficient mice may be related to the increased apoptosis of megakaryocytes. These data suggest that the apoptosis pathway inhibited by Bcl-xl should be hindered during thrombopoiesis (**Figure 4**).

It is reported that thrombocytopenia is the most commonly side effect of clinical Bcl-xl inhibition (151). Bcl-xl inhibition not only led to platelet death, but also negatively affected CD61^+^CD41^+^ cell survival (152). Furthermore, Afreen et al. found that the development of MKs was negatively affected by the inhibition of Bcl-xl, which was demonstrated by the finding that continuous inhibition of Bcl-xl seriously affected the production of platelets from mice or CD34^+^ cells *in vitro* (152). Taken together, these data suggest that when MKs reach the platelet shedding point of PPF, they become dependent on Bcl-xl. In other words, caspase activation has a negative impact on the ability of MKs to produce platelets.

Bak and Bax are key mediators of the intrinsic apoptotic pathway. Given the putative role of the intrinsic apoptotic pathway in platelet production, researchers have used genetic approaches to analyze the consequences of blocking or activating MK apoptosis. A mouse model with Bak structural deletion and Bax-MK-specific deletion was developed. The combined deficiency of Bak and Bax makes a variety of cell types resistant to physiological and pathological inducers of apoptosis (153). Surprisingly, Josefsson et al. found that Bak^-/-^Bax^Pf4Δ/Pf4Δ^ mice showed normal MEP countand serum TPO levels. The megakaryopoiesis and PPF were not affected in Bak^-/-^Bax^Pf4Δ/Pf4Δ^ mice. These results indicate that Bak and Bax have no effects on megakaryocytopoiesis and thrombopoiesis (149). However, the platelet count in Bak^-/-^ mice was significantly higher than that in wild-type mice. And with the additional loss of Bax, the platelet count remained elevated (149). Given the role of these two proteins in regulating platelet lifespan, the authors further investigated the kinetics of platelet clearance *in vivo*. As previously reported, platelet lifespan was significantly prolonged in Bak^-/-^ and Bak^-/-^ Bax^Pf4Δ/Pf4Δ^ mice (149). These data confirm that megakaryocytopoiesis and thrombopoiesis do not require Bax- and Bak-induced apoptosis and that inhibition of intrinsic apoptosis leads to a significant increase in platelet lifespan, and consequently increasing the number of platelets (**Figure 4**).

Sim et al. identified different stages of MK differentiation based on the phenomenon that the size and granularity of MKs increased with MK maturation. Immature LG MKs developed into mature HG MKs, which were further split into a functional CD42b^+^ population and an apoptotic, nonfunctional CD42b^-^ population (154). CD42b surface expression is a critical indicator for assessing platelet quality since metalloproteinase cleavage occurs after activation, apoptosis, or inappropriate storage (155). Q-VD-Oph, an effective pan-caspase inhibitor, reduced apoptosis as well as CD42b shedding, experiencing a decline in the percent of apoptotic CD42b^-^ MKs and an increase in the percentage of CD42b^+^ MKs (154). Furthermore, the authors observed an increase in platelet production *in vivo* after infusion of Q-VD-Oph-treated MK, but a decrease in PLP production *in vitro* for the same MK (154). This may be due to differences in thrombopoiesis *in vitro* and *in vivo*. Inhibition of apoptosis can preserve mature and platelet-producing MKs, and increase the production of platelets *in vivo*.

Although FasL induces an extrinsic apoptosis pathway in MKs, DR2 activation does not stimulate platelet production but triggers caspase 8-mediated apoptosis (131). In addition, neither caspase 8 deletion nor combined caspase 8/Bak/Bax deletion affects platelet production (131). Instead, this protects MKs from death in mice infected with the lymphocytic chorionic meningitis virus (156). Moreover, it had been reported that hematopoietic mice lacking Apaf-1 and Cyt c had normal platelet count (131). These results suggest that apoptosis is dispensable for the thrombopoiesis and should even be inhibited. In other words, apoptosis has a neutral or inhibitory effect on thrombopoiesis (**Figure 4**).

Collectively, apoptosis has a negative impact on the ability of MKs to produce platelets, which runs counter to the previous studies that MKs require apoptotic caspases to promote thrombopoiesis and platelet shedding.

## 5 Other more limited and specific apoptosis-like processes may be involved in thrombopoiesis

MKs do not require Bak/Bax-mediated intrinsic apoptosis signals and caspase 8-dependent extrinsic apoptosis pathways to produce platelets. However, they may experience limited or specific apoptosis-like processes and caspase 8-mediated downstream apoptosis mechanisms, which are independent of Bak and Bax activation. This may involve their activation through the non-classical apoptotic signaling pathways or their non-cell death-related functions. Alternatively, PPF can be induced by other programmed cell death pathways, such as autophagy, instead of the classical caspase-dependent apoptosis (157). Autophagy is a highly conserved, lysosome-mediated catabolic process that allows cells to degrade unwanted cytoplasmic components and recovers nutrients in a regulated manner (158). It is accomplished by a series of autophagy-related genes (ATGs) (159). Autophagy plays an important role in maintaining the stemness and microenvironment of HSCs (160, 161). In the past few decades, the multipotent roles of autophagy in blood cell development have been demonstrated in erythropoiesis and hematologic malignancies (162, 163). Dysfunction of autophagy disrupts the proliferation and differentiation of myeloid progenitor cells (164–166). Sun et al. find that an inducer of autophagy, rapamycin, promotes MK maturation and thrombopoiesis without affecting platelet function. Conversely, bafilomycin A1, an inhibitor of autophagy, inhibits thrombopoiesis (167). Cao et al. find that ATG7-deficient mice exhibit abnormal megakaryocytopoiesis, MK differentiation, thrombopoiesis, and hemostasis (168).

## 6 Discussion

Is apoptosis a safeguard or a threat in thrombopoiesis? We tried to get in an accurate answer to the question, but struggled. Since the late 1990s, there has been considerable evidence that the apoptosis has a positive effect on thrombopoiesis, which activate MKs to promote PPF and platelet shedding. However, the exact mechanism underlying this pathway has not yet been elucidated. Recent studies have shown that MKs have a functional intrinsic apoptotic pathway, but this is not required for thrombopoiesis and is inhibited during thrombopoiesis. We comprehensively consider the research status of the mechanism of apoptosis and thrombopoiesis, and we do believe that time needs to be spent on robust structured research to answer the unanswered questions in this interesting and expanding field. There are several factors that can explain the difference in the above results.

(1). Several of the key studies reporting apoptosis and platelet production have involved overexpression or knockdown of apoptosis-related proteins, either in mice or cell cultures. Different technical means may affect the results, such as structural knockout or conditional knockout of genes. Overexpression or knockdown of any protein can affect cellular processes in complex and unpredictable ways, especially *in vivo*.
(2). The interactions between individual apoptotic molecules have rarely been examined. Each molecule in a biological process does not exist singularly or act alone, and further studies are needed to determine whether they are functionally redundant or interact with each other.
(3). Bax and Bak may have additional non-canonical functions (169). Bax triggers nuclear protein redistribution (NPR) when apoptosis is stimulated or Bax is forcibly expressed in the nuclear envelope (NE) (170). This process involves the disruption of NE proteins regulated by Bax, including laminin A/C, resulting in the creation and subsequent rupture of bubbles containing nucleoproteins that are wrapped in NE depleted of nuclear pores. This process, known as stress-induced nuclear bubble production and rupture (SIGRUNB), results in the release of nuclear proteins into the cytoplasm. It precedes the morphological changes of apoptosis, is independent of the release of caspase and Cyt c, and is not inhibited by Bcl-xl (171). This may also cause Bak/Bax activation ultimately leading to impaired thrombopoiesis or lifespan.
(4). The number of platelets is maintained in a dynamic and stable range, which is inseparable from the development of the megakaryocytic lineage, platelet production and platelet clearance/lifespan, which should be measured comprehensively by the relevant organ and physiological indicators. For example, the lung is also one of the organs where platelets are produced.
(5). Results from *in vitro* culture systems, cell lines, and chemical inhibitors should be treated with caution. The study of *in vitro* culture system or cell line can’t comprehensively evaluate the clearance/lifespan of platelets, which is a serious defect in the study of platelet number homeostasis. The results of chemical inhibitors should be carefully analyzed. For example, both zVAD.fmk and Q-VD-OPh are broad-spectrum caspase inhibitors. However, high concentrations of the zVAD.fmk inhibit proplatelet formation in MKs, but Q-VD-OPh did not (149). This may be because high concentrations of zVAD.fmk are toxic to MKs and caspase-independent. In fact, zVAD.fmk has been clearly reported to have potent activity against histones B, H and L (172), leading to necrosis in some cells.
(6). The studies about the role of apoptosis in thrombopoiesis were scattered and not systematic. The most studies only showed the phenotypes of knockout or overexpression mice or cells of apoptosis-related genes. Whether the expression change of apoptosis-related genes can affect the factors or signaling pathways involved in megakaryocyte production, differentiation and maturation, proplatelet formation or platelet generation is not deeply explored. The clues provided by existing researches are very limited. Therefore, the truth behind this fact is still not clear for us. The relationship of signaling pathways between apoptosis and thrombopoiesis is worthy of further in-depth study and their gene regulatory network should be gradually established.
(7). Currently, the vast majority of studies on the role of apoptosis in the dynamic maintenance of platelet count are still at the stage of mouse or *in vitro* studies. Zebrafish should also be considered as a research model. The similarities between zebrafish and mammals in platelet developmental and physiological processes suggest that advances in zebrafish research will reveal an understanding of human platelet-related biology and disease (173).
(8). Finally, the occurrence of clinical disease has multiple causes. Based on clinical studies and other basic preclinical studies, it is doubtful that thrombopoiesis relies on apoptosis in the traditional sense. For example, chemotherapeutic drugs play an antitumor role by inducing apoptosis in tumor cells. Many chemotherapeutic agents and regimens can cause thrombocytopenia (174). Although there are different causes of thrombocytopenia, including insufficient platelet production, excessive platelet destruction, and sequestration of platelets in the spleen, the major cause of low platelet counts in cancer is the inhibition of platelet production in patients receiving chemotherapy. For instance, carboplatin is a metal complex that binds to DNA in the nucleus through multiple steps to form a structure which disrupts the biological function of DNA, prevents DNA replication, and thereby inhibits cancer cell growth or promotes apoptosis of cancer cells (175, 176). This process is also accompanied by the death of MKs and the occurrence of thrombocytopenia. Furthermore, some pathogens, including arenaviruses (177), HIV (178), dengue (179), and anthrax (180) can cause MK apoptosis, leading to thrombocytopenia. In addition, myelodysplastic syndrome (181) and immune-mediated thrombocytopenia (181) are associated with MK apoptosis. In patients with idiopathic thrombocytopenic purpura, MKs show characteristics of apoptosis-like programmed death (182). However, multiple concomitant mechanisms contribute to low platelet counts in patients with immune thrombocytopenia. Desialylation and apoptosis may be relevant mechanisms leading to platelet destruction (183).

In conclusion, targeting apoptosis in hematopoietic system precision medicine is certainly a very attractive strategy. Whereas the apoptotic pathway may display a therapeutic candidate to treat platelet number abnormality related diseases, there is an urgent need to improve our understanding of the contribution and the underlying molecular mechanisms of the apoptotic pathway to (stress) platelet production in human in order to develop new opportunities for the treatment of platelet number abnormality related diseases. A comprehensive analysis of apoptosis-related molecules, the regulatory mechanism of MKs and thrombopoiesis is of great practical significance for treating platelet-related diseases (**Table 1**). There may be other limited or specific apoptotic processes that are tightly and precisely controlled during platelet formation. This knowledge may be useful for future *in vitro* platelet dilation or *in vivo* therapy to improve thrombopoiesis in patients with thrombocytopenia. In addition, a better understanding of the biological process and molecular mechanisms by which MKs produce platelets may help to guide the development of new therapies to treat the various forms of thrombocytopenia. Moreover, further study of apoptosis will provide new therapeutic targets and develop new drugs to treat platelet-related diseases.

**Table 1:** The subtle relationship between thrombopoiesis and apoptosis-related molecules.

Apoptotic molecules (Abbreviation)	Major function	Model	Details	Result	Refs
Positive effects of apoptosis on thrombopoiesis.					
Bcl-2	Anti-apoptotic protein	Mice	Expressing human Bcl-2 in mice	Damage the formation of PPF. Decrease the number of peripheral blood platelets.	(128)
Bim	Pro-apoptotic protein	Mice	Bim knockout mice	The number of peripheral blood platelets decreased.	(129)
Bcl-xl	Anti-apoptotic protein	Mice	Bcl-xl overexpression mice	Proplatelet formation is impaired.	(130)
FasL	Pro-apoptotic protein	Cell	Fas ligand (FasL) or agonistic Fas antibodies were added to meg01 cells or mouse derived megakaryocyte culture system	Platelet-like particles increased significantly.	(116)
TRAIL	Pro-apoptotic protein	Cell	Recombinant TRAIL protein was added into the CD34^+^ cells culture system	The apoptosis rate increased significantly, and promoted megakaryoblasts into fully mature platelet producing megakaryocytes.	(132)
Caspase 8	Pro-apoptotic protein	Cell	Caspase 8 was added to the culture system	The number of platelets doubled from before.	(131)
Caspase	Pro-apoptotic protein	Cell	A broad-spectrum caspase inhibitor, z-VAD-fmk was added into the CD34^+^ cells culture system	Proplatelet and functional platelets decrease significantly.	(131)
Neutral or inhibitory effects of apoptosis on thrombopoiesis.					
Bcl-2	Anti-apoptotic protein	Mice	Bcl-2 knockout mice	Normal platelet count	(146)
Mcl-1	Anti-apoptotic protein	Mice	Mcl-1 knockout mice	Normal platelet count	(146)
BCL2L2	Anti-apoptotic protein	Cell	CB derived MK overexpressing BCL2L2	Proplatelet formation is increased.	(148)
Apaf-1	Pro-apoptotic protein	Mice	Apaf-1 knockout mice	Normal platelet count	(131)
cytochrome C	Pro-apoptotic protein	Mice	cytochrome C knockout mice	Normal platelet count	(131)
Caspase 8	Pro-apoptotic protein	Mice	Caspase 8 knockout mice	Normal platelet count	(131)
Bcl-xl	Anti-apoptotic protein	Mice	Bcl-xl knockout mice	80% of Bcl-xl knockout mice suffer from severe thrombocytopenia, and their ploidy and serum TPO levels decrease significantly.	(149)
BH3-only protein	Pro-apoptotic protein	Cell	ABT-737 was added into bone marrow-derived MKs culture system	ABT-737 inhibits PPFs in a dose-dependent manner.	(184)

## Author contributions

All authors contributed to the article and approved the submitted version. SY, LW and JW provided the main plan and conceptual ideas. The first draft of the manuscript was written by SY, LW, YW, AW, FH and XT reviewed and made significant revisions to the manuscript. FK and SA, DQ, QM, JC, XH, and CZ revised the manuscript. JW provided direction and guidance throughout the preparation of this manuscript.
